# Performance of the new biological small- and wide-angle X-ray scattering beamline 13A at the Taiwan Photon Source

**DOI:** 10.1107/S1600576722001923

**Published:** 2022-03-18

**Authors:** O. Shih, K.-F. Liao, Y.-Q. Yeh, C.-J. Su, C.-A. Wang, J.-W. Chang, W.-R. Wu, C.-C. Liang, C.-Y. Lin, T.-H. Lee, C.-H. Chang, L.-C. Chiang, C.-F. Chang, D.-G. Liu, M.-H. Lee, C.-Y. Liu, T.-W. Hsu, B. Mansel, M.-C. Ho, C.-Y. Shu, F. Lee, E. Yen, T.-C. Lin, U. Jeng

**Affiliations:** a National Synchrotron Radiation Research Center, Hsinchu Science Park, Hsinchu 30076, Taiwan; b Institute of Biochemical Sciences and Institute of Biological Chemistry, Academia Sinica, Nankang, Taipei 11529, Taiwan; c Academia Sinica Grid Computing Centre, Academia Sinica, Nankang, Taipei 11529, Taiwan; d Department of Chemical Engineering, National Tsing Hua University, Hsinchu 30013, Taiwan

**Keywords:** small-angle X-ray scattering, wide-angle X-ray scattering, SAXS–WAXS, SWAXS, online size exclusion chromatography, integrated UV–Vis absorption and refractometry, biomolecular solution scattering

## Abstract

Recent developments in the instrumentation and data analysis of synchrotron small-angle X-ray scattering (SAXS) on biomolecules in solution have made biological SAXS (BioSAXS) a mature and popular tool in structural biology. This article reports on an advanced endstation developed at beamline 13A of the 3.0 GeV Taiwan Photon Source for biological small- and wide-angle X-ray scattering (SAXS–WAXS or SWAXS). The endstation features an in-vacuum SWAXS detection system comprising two mobile area detectors (Eiger X 9M/1M) and an online size-exclusion chromatography system incorporating several optical probes including a UV–Vis absorption spectrometer and refractometer. The instrumentation and automation allow simultaneous SAXS–WAXS data collection and data reduction for high-throughput biomolecular conformation and composition determinations. The performance of the endstation is illustrated with the SWAXS data collected for several model proteins in solution, covering a scattering vector magnitude *q* across three orders of magnitude. The crystal-model fittings to the data in the *q* range ∼0.005–2.0 Å^−1^ indicate high similarity of the solution structures of the proteins to their crystalline forms, except for some subtle hydration-dependent local details. These results open up new horizons of SWAXS in studying correlated local and global structures of biomolecules in solution.

## Abstract 2

A new endstation for biological small- and wide-angle X-ray scattering is detailed, which provides development opportunities for studying correlated local and global structures of biomolecules in solution.

## 1. Introduction

Capable of revealing overall shapes and structural transitions of biological macromolecules in solution, biological small-angle X-ray scattering (BioSAXS) is increasingly used in structural biology. Recent reviews (Schroer & Svergun, 2018 ▸; Jeffries *et al.*, 2021 ▸; Bizien *et al.*, 2016 ▸; Trewhella *et al.*, 2017 ▸) summarize the state of the art in BioSAXS and project its future developments and applications. Currently, BioSAXS contributes significantly in validating protein conformations in solution based on the structures reconstructed from protein crystallography, NMR, electron spin resonance, cryoEM, small-angle neutron scattering or molecular simulations, to name but a few (Schroer & Svergun, 2018 ▸). Furthermore, BioSAXS data analysis can combine information from partial structures obtained from different techniques to build a conformation of a full protein or protein complex, including adding missing local domains or flexible loops; the latter is particularly relevant to intrinsically disordered proteins and protein folding–unfolding intermediates (Bizien *et al.*, 2016 ▸; Bernadó *et al.*, 2007 ▸).

However, protein solution small-angle X-ray scattering (SAXS) often encounters difficulties from weak scattering intensity, protein aggregation and radiation damage. Low scattering background (less than *ca* 10^−4^ cm^−1^, *i.e.* a few per cent of the background scattering from the aqueous solvent used) and reliable background scattering measurements and subtraction are essential requirements for synchrotron BioSAXS endstations to retrieve the notoriously weak scattering of proteins in dilute solution. Recently, online size-exclusion chromatography (SEC) has become nearly a standard in BioSAXS (Jensen *et al.*, 2010 ▸; Ryan *et al.*, 2018 ▸; Cowieson *et al.*, 2020 ▸), allowing *in situ* kinetic separation of aggregation and coexisting species (Shih *et al.*, 2017 ▸, Yeh *et al.*, 2017 ▸) in a sample solution followed by SAXS data collection. In particular, sample flow in SEC–SAXS measurements greatly alleviates the synchrotron radiation damage that is commonly encountered (Jeffries *et al.*, 2015 ▸; Castellví *et al.*, 2020 ▸). Recent pioneering BioSAXS endstations also incorporate optical measurements of UV–Vis absorption and refractive index (RI) (Blanchet *et al.*, 2015 ▸); in some cases, multi-angle light scattering (MALS) and dynamic light scattering (DLS) are further integrated along the SEC–SAXS sample elution path for concentration, composition and mass determination of proteins or protein complexes (Graewert *et al.*, 2020 ▸) in one sample elution. In particular, incorporating RI measurements allows better signal monitoring and background scattering subtraction from the coexisting detergent micelles (which show no UV–Vis absorption) in SEC–SAXS measurements with membrane proteins stabilized by detergents in solution (Berthaud *et al.*, 2012 ▸).

BioSAXS data analysis methods were reviewed in detail recently (Bizien *et al.*, 2016 ▸; Schroer & Svergun, 2018 ▸), including *ab initio* methods for low-resolution shapes and hybrid modeling to assemble semi-atomic structural models and atomistic structures (Manalastas-Cantos *et al.*, 2021 ▸). The ensemble optimization method has also been developed to resolve polydisperse conformations of intrinsically disordered proteins from a SAXS profile (Schroer & Svergun, 2018 ▸; Sagar *et al.*, 2021 ▸; Tria *et al.*, 2015 ▸). With improved instrumentation and data quality, the current SAXS data analysis methodology faces the challenge of handling data from wide-angle X-ray scattering (WAXS), but collecting and interpreting BioWAXS data are both tough tasks. As illustrated with all-atom mol­ecular dynamics simulations (Knight & Hub, 2015 ▸; Hub, 2018 ▸; Schroer & Svergun, 2018 ▸), resolving small structural features in the hydration layer from the highly convoluted and broad WAXS humps consumes computation resources greatly. Experimentally, separating the very weak WAXS intensity of a dilute protein solution from the background scattering of the solvent and air-path molecules (Yang *et al.*, 2020 ▸) requires a dedicated WAXS detection environment.

To meet all the challenges mentioned above, a dedicated biological small- and wide-angle X-ray scattering (BioSWAXS) beamline (13A) at the 3.0 GeV Taiwan Photon Source (TPS) has been jointly developed by the National Synchrotron Radiation Research Center (NSRRC) and Academia Sinica. The beamline, open to users since September 2020, features an in-vacuum SWAXS detection system and an online size-exclusion system incorporating several optical probes. The illustrated SWAXS data measured for several model proteins show clear global and local structural features of the proteins in solution, hence differentiating their fully hydrated structures from the corresponding crystalline structures. With its unique instrumentation and automation, the new TPS 13A BioSWAXS endstation offers new development opportunities to resolve challenging questions and initiate new fields in structural biology research.

## 2. Beamline overview

Equipped with a 4 m in-vacuum undulator (IU24) (consisting of 168 magnets arranged in a period length of 24 mm), the TPS 13A BioSWAXS beamline provides high-brilliance X-rays in the energy range from 4.0 to 23 keV. The beamline comprises four major zones – front-end (21.2 m), optical systems (15.5 m), endstation hutch (18.1 m) and data acquisition operation (4.7 m) – as outlined in Fig. S1 of the supporting information. The beamline optics and performance were detailed previously (Liu *et al.*, 2021 ▸). Briefly, the major optical components in the optical zone are an integrated double-crystal monochromator (DCM) composed of Si(111) single crystals and a double-multilayer monochromator (DMM) composed of Mo/B_4_C multilayers, selectively used in the high-flux or high-energy-resolution mode. These are followed by two sets of vertical/horizontal Kirkpatrick–Baez (K-B)-type focusing mirrors (VFM/HFM) for an optional focus at the sample or the detector position (40 or 52 m from the source). A horizontally oriented four-bounce double-crystal collimator (4BCC) situated at the end of the optical zone can be selectively moved into the beam path for ultra-small-angle X-ray scattering (USAXS) without altering the downstream beam path, and moved out of the X-ray path simply by rotating the two sets of the crystal collimator to be parallel with the X-ray beam. The 4BCC consists of two sets of Si(311) double-crystal collimators oriented in the horizontal diffraction plane and arranged in a dispersive configuration to significantly decrease the beam divergence to *ca* 30 µrad. This could effectively trim down an X-ray beam profile by nearly 100 times less background scattering intensity, albeit with a tenfold loss of the main beam intensity (Liu *et al.*, 2021 ▸).

This new beamline features four characteristic operation modes for advanced structural studies on biomolecules: (1) The high-flux mode with the DMM provides up to 4 × 10^14^ photons s^−1^ in the energy range of 7–15 keV at the sample area. The high flux and the fast Eiger X 9M (500 Hz frame rate) and X 1M (3 kHz frame rate) detectors, respectively, for SAXS and WAXS equip the beamline for time-resolved SWAXS measurements with millisecond resolution. (2) The anomalous SWAXS mode with the DCM provides X-rays of a general energy resolution of 0.02% in an energy range of 4–23 keV, covering from the *K* edge of calcium (4028.1 eV), which is of biological importance, to that of ruthenium (22117.2 eV), important for catalytic applications. (3) The microbeam SWAXS mode is enabled with a microslit system and the sample-focusing K-B mirrors moved into the beam path for an X-ray beam size down to *ca* 10 µm at the sample position. (4) The USAXS mode with the DCM and 4BCC covers a very low scattering vector magnitude *q* down to 0.0003 Å^−1^, with a low beam energy (4.0 keV) and a long sample-to-detector distance (10 m).

## 3. Endstation

**Figure 1:**
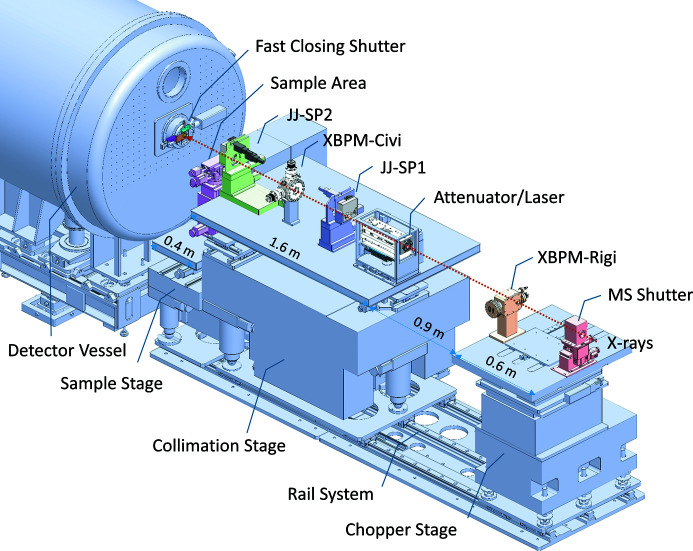
Key components along the X-ray path before the sample position (40 m from the X-ray source IU24). From upstream, there are millisecond shutters (MS shutter) and the first set of X-ray beam position and intensity monitors (XBPM) called Rigi, sitting on the granite chopper stage (to accommodate a future chopper system for time-resolved measurements). These are followed by an attenuator, the first set of JJ slits/pinholes (JJ-SP1), the second set of XBPM (Civi) and the second set of JJ slits/pinholes (JJ-SP2) sitting on the collimation granite stage. A motorized PIN diode (5 mm-diameter active area), situated 1 mm after the S_3_N_4_ vacuum exit window, can be moved into the beam path for centering the JJ-SPs.

The TPS 13A endstation consists of three major systems: collimation, sample and detection. The first two systems sit on two separate granite stages that can translate along the X-ray path on the same rail system (Fig. 1 ▸) with their positions defined by the encoder system of a magnetic ruler (Renishaw). This design allows convenient tuning of the sample area space to accommodate different sample environments.

### 3.1. Collimation system

The pre-sample zone in the endstation has (along the X-ray path) a millisecond (MS) shutter, two sets of X-ray beam position and intensity monitors (XBPMs, termed Rigi and Civi), an attenuator, a laser alignment system, and two sets of JJ X-ray slits/pinholes (JJ-SP1 and JJ-SP2) (Fig. 1 ▸). The pre-sample vacuum region is terminated by a 1 µm-thick, 10 × 10 mm S_3_N_4_ vacuum window, followed by a photodiode that can be moved in and out to position the JJ-SP at the beam center. The major beam collimation is controlled by four tungsten-based blade slits (typical opening of 0.3 × 0.2 mm in horizontal and vertical directions) sitting at the 31.65 m position in the optical zone and JJ-SP1 (39.2 m position) in the endstation. JJ-SP2, placed after Civi, serves as the guard slits. The stage of JJ-SP2 can be translated along the X-ray path to minimize the air path before the sample position at 40 m from the X-ray source. Four Ta pinholes with diameters 0.15, 0.20, 0.3 and 0.4 mm are embedded on each of the two vertical blades of JJ-SP1 and JJ-SP2 for an option of pinhole collimation. The two stages of the JJ-SP sets allow fine titling (in two dimensions) of the slit blades or pinhole on the scale of µrad for ideal normal incidence of the X-ray beam, which proves to be effective in reducing parasitic slit/pinhole edge scattering.

With in-house electric current reading systems and voltage-to-frequency converters, the two four-quadrant-diamond XPBMs Rigi and Civi (respectively, with 20 and 3 µm gaps) in the beam collimation section report beam intensity and position with 1 µm resolution. The X-ray-irradiation-induced electric current detected by Rigi (located before the attenuator) is used for intensity normalization in transmission measurements, with a highly attenuated direct beam (with and without sample, respectively) recorded by the SAXS detector. The X-ray-induced electric current recorded by Civi (located after the attenuator and JJ-SP1) is used in normalizing the incident beam intensity in SWAXS measurements.

### 3.2. Sample environment

**Figure 2:**
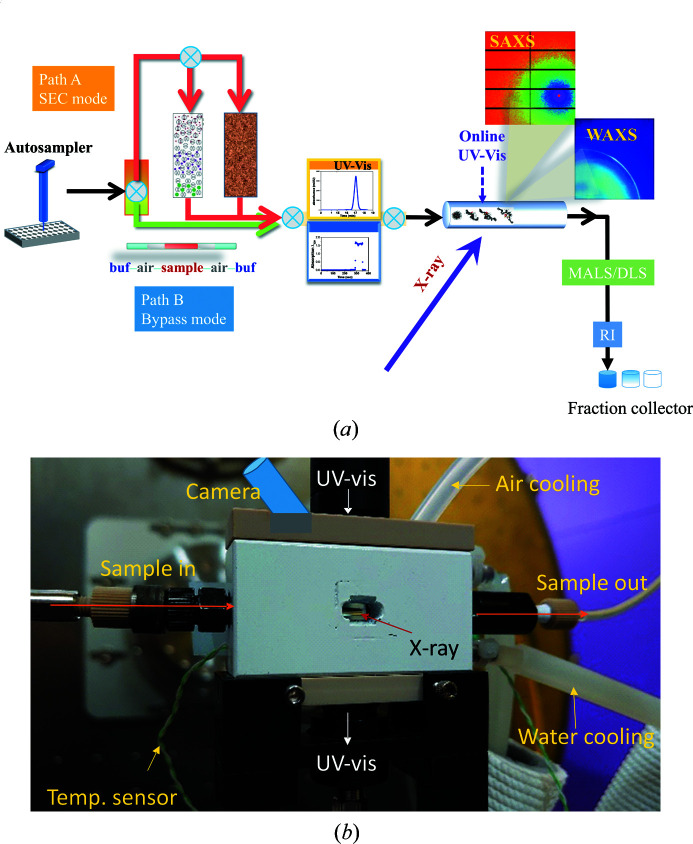
(*a*) Schematic diagram of the TPS 13A online two-column SEC–SWAXS/UV–Vis/MALS/DLS/RI system, based on an Agilent 1260 HPLC sample purification system. An autosampler injects the samples into the system. Path A leads the sample to a SEC column, whereas Path B bypasses the column. After the HPLC UV–Vis absorption detection, the sample flows into a quartz capillary cell for simultaneous UV–Vis absorption and SWAXS measurements on the same sample spot. The elution continues to optional MALS and DLS measurements, and then a default RI detection before fraction sample collection. (*b*) A close-up photograph of the quartz capillary housing, with the pathways of the sample, camera, UV–Vis light, and air and water cooling indicated. The X-ray beam is indicated by a red arrow

A sample stage with ten degrees of freedom is installed on a large granite stage with three degrees of freedom (Fig. 1 ▸). Various sample environments can be set up on the sample stage according to specific requests in sample controls for SWAXS measurements. Specifically, Linkam cells THMS600 for high-temperature controls and TST350 for accurate tensile characterization with temperature and humidity controls are available. Below, we elaborate on the SEC–SWAXS system dedicated to biomolecular solutions. The design of the unique TPS 13A SEC system is based on an Agilent chromatographic system (1260 series) for high-performance liquid chromatography (HPLC). The system is integrated with UV–Vis absorption detection at the X-ray sample position, SWAXS experiments, optional MALS with one of 18 angles replaced by DLS measurements, and RI detection before fraction sample collection. Fig. 2 ▸(*a*) shows a schematic diagram of SEC–SWAXS at the TPS 13A endstation. Three flow pumps, four six-way valves and one ten-way valve are installed in the SEC system to form a two-column system. As a result, one column system can deliver the sample for SWAXS measurements while the other is under cleaning or equilibrium with a different buffer (detailed in Fig. S2). In addition to the two-column SEC mode, an optional SEC-bypass flow mode is also available to better preserve the sample concentration for SWAXS. After each sample elution, a washing mode is activated to clean the sample quartz capillary (Fig. S3). Moreover, the Agilent 1260 system provides an online sample-mixing mode for *in situ* measurements. Three types of columns are available at the TPS 13A endstation: (1) Agilent Bio SEC-3 silica-based columns (pore size 100, 150 and 300 Å for molecular-weight ranges of 0.1–100, 0.5–150 and 5–1250 kDa, respectively), (2) GE Superdex 200 Increase 5/150 GL polymer-based columns (for highly charged proteins), and (3) TSKgel G6000PWXL columns (Tosoh Bioscience GmbH) for large molecules up to 8000 kDa.

A standard SEC–SWAXS operation at the endstation starts from the autosampler, which is a remote-controlled pipette that can execute programmed sampling from the samples preloaded in a thermostated 99-sample tray and subsequent injection into the SEC system. The operation is followed by the HPLC UV–Vis detection, as shown in Fig. 2 ▸(*a*). The sample subsequently flows through a thin-wall (10 µm) quartz capillary with a typical diameter of 2.0 mm [Fig. 2 ▸(*b*)], illuminated with a 15 keV beam. To cover data in a lower *q* range, a lower-energy beam of *e.g.* 12 or 8 keV can be used; correspondingly, a 1.5 or 1.0 mm capillary is used to compensate for the transmission loss. The X-ray beam (of dimensions *ca* 200 × 300 µm) and a UV–Vis light beam (*ca* 1 mm diameter) irradiate the sample flow at the same zone on the capillary cell (in orthogonal configuration) for SWAXS and *in situ* UV–Vis absorption measurements (190–1000 nm). The capillary cell housing block has a water circulation system to control the ambient temperature around the capillary (273–353 K). A dry, cool airflow (same temperature as the buffer solution) towards the capillary prevents water condensation during a low-temperature measurement. The air-cooling system also dissipates the heat on the capillary hot spot from X-ray irradiation. A small video camera (3IG Corporation Limited, model IPM604W530LB) is installed on the capillary housing block to check the sample flow. After the capillary, the sample continues flowing through a differential refractometer (Optilab T-rEX) 30 cm downstream for refractive index measurement and then finishes its journey by the fraction collector for sample recycling. A MALS–DLS system can be selectively connected to the elution path between the sample capillary and RI detection for protein mass and hydro­dynamic radius measurements.

### 3.3. X-ray detection

The X-ray detection at the TPS 13A includes the primary scattering image detection system comprising an Eiger X 9M detector for SAXS and an Eiger X 1M detector for WAXS. A silicon drift detector (SDD) and the two XBPMs (Rigi and Civi) are used for sample positioning, beam location, energy recording and intensity monitoring. All these work in synchrony in the SWAXS data collection.

#### 3.3.1. Detector vessel

**Figure 3:**
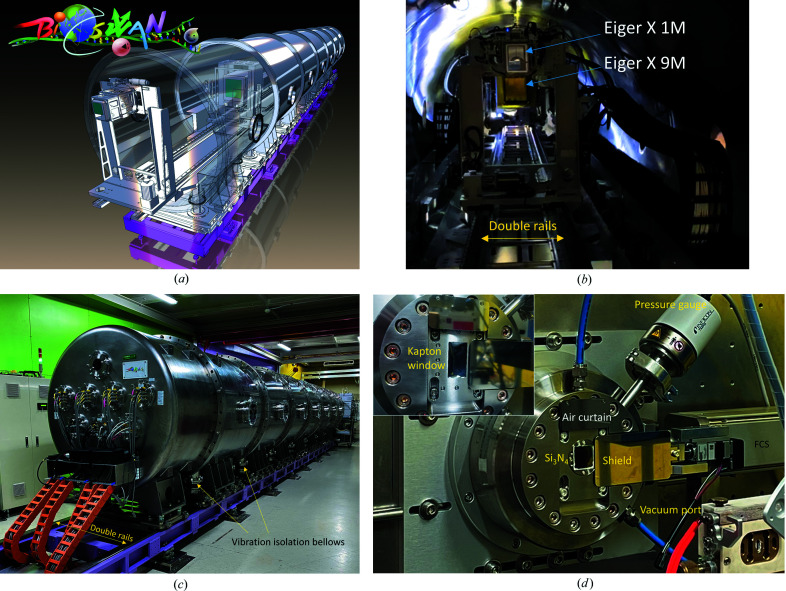
(*a*) Cartoon illustration of the vacuum vessel for the SWAXS detection system at the TPS 13A endstation. (*b*) Inside the vacuum vessel, the Eiger X 1M (WAXS detector) sits on its stage, rolling on the rail system at the front; the Eiger X 9M (SAXS detector) is farther on the same rail system. (*c*) A back view of the detector vessel, with all the vacuum feedthroughs on the end tank. The detector vessel sits on another double-rail system to translate the whole system along the X-ray beam. Vibration isolation bellows (indicated) are installed to reduce the effects of detector vessel deformation during vacuum pumping on the precision rail alignment of the detector double-rail system enclosed inside the vacuum vessel. (*d*) Photograph of the entrance window and fast-closing shutter (FCS) selectively sealed with a Kapton window (40 mm by 60 mm) or an Si_3_N_4_ window (15 mm by 15 mm). Also shown are a protection shield for the entrance window and an air curtain to dispel hot air from a heated sample stage close to the window.

A 12 m-long vacuum vessel with a diameter of 1.5 m was designed to accommodate the two SAXS–WAXS detectors [Fig. 3 ▸(*a*)]. The detector vessel comprises seven straight 1.5 m sections, one front cap (0.3 m) and one end tank (0.85 m). Every 1.5 m-long section is embedded with double rails inside to be assembled into an 11.55 m translation rail system for the two Eiger detectors [Fig. 3 ▸(*b*)]. The whole detector vessel sits on top of another base rail system fixed on the endstation floor, which can translate the whole 12 m vessel along the X-ray path as one unit to tune the sample area’s space. Moreover, each 1.5 m-long section can be motorized to open and close for detector installation and maintenance. The end-cover tank of the detector vessel provides vacuum feedthroughs for all signal and power cables and can be withdrawn for 800 mm to load the Eiger X 9M SAXS detector [Fig. 3 ▸(*c*)]. Loading and maintenance of the Eiger X 1M WAXS detector are conducted by opening the front cap of the detector vessel. A position encoder system comprising a Renishaw magnetic ruler is installed on the rail system of the two Eiger detectors for reading their absolute positions with sub-millimetre accuracy.

The front cap of the vacuum vessel has a 100 mm-diameter opening sealed by a fast-closing shutter (FCS) (VAT Group AG), as shown in Fig. 3 ▸(*d*). The FCS connects to an upstream adaptor with an X-ray entrance window made of 1.5 µm-thick Si_3_N_4_ (15 × 15 mm) or 12 µm-thick Kapton (40 × 20 mm). An in-house-made FCS controller allows triggering of the FCS to close within 3 ms when detecting a significant pressure difference between the two pressure gauges near the entrance window and the middle section of the detector vessel, due to an unexpected window failure. The detector vessel is pumped down to less than 10 mTorr (1 Torr = 133.322 Pa) within 2–3 h by a pumping system comprising three dry screw vacuum pumps (two Edward GX600L and one GX100L), and then maintained below 10 mTorr (with one of the two Edward GX600Ls off) for the working conditions of the two Eiger detectors. A graphical user interface (GUI) was constructed to start a programmed evacuation or venting process with regulated pumping speeds to reduce the risk of the thin entrance window failing because of unexpectedly abrupt pressure changes.

#### 3.3.2. SAXS detector

**Figure 4:**
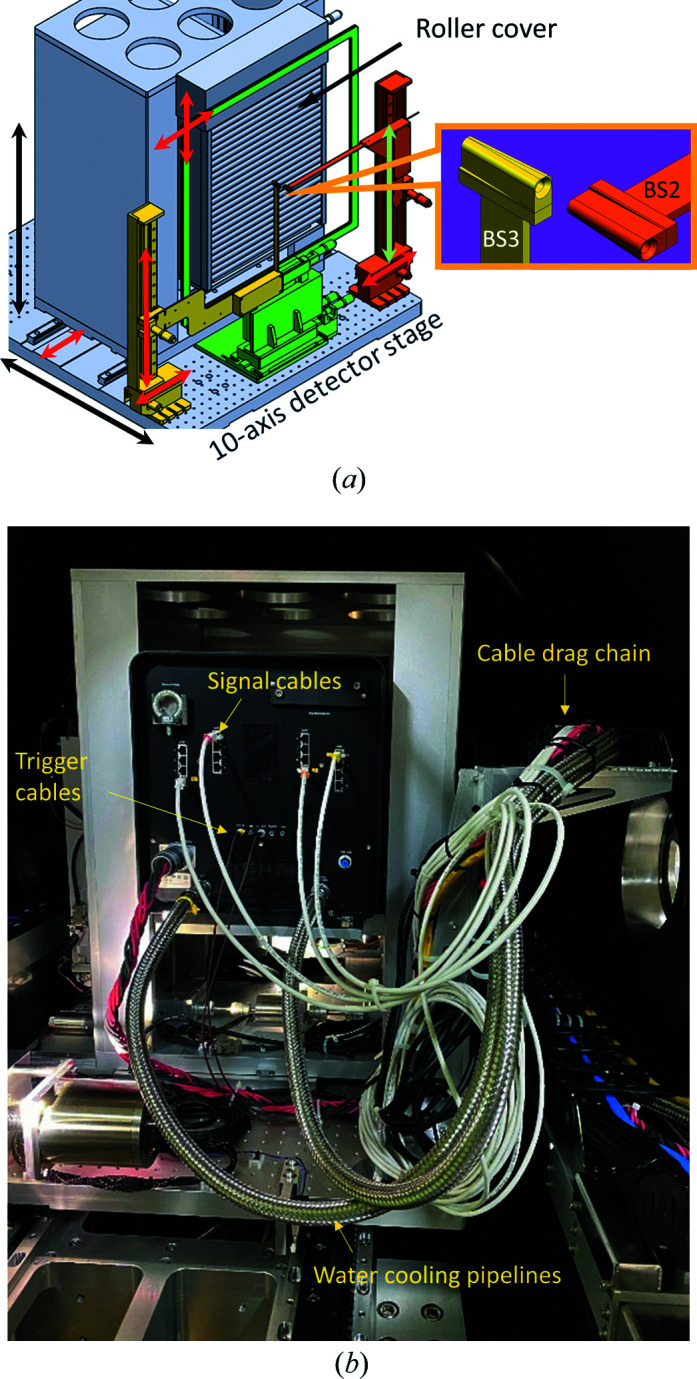
(*a*) Schematic diagram of the Eiger X 9M SAXS detector stage with ten degrees of freedom as indicated by the ten arrows, including ±120 mm detector translation in the vertical and horizontal directions. There is an in-house-made motorized aluminium roller to cover and protect the detector active area when needed. A Ta beamstop (BS1) is glued on the center of a Kapton film of 8 µm thickness on the green frame (±20 mm in vertical and horizontal directions). The other two beamstops (BS2 and BS3, orange and yellow) are each equipped with a photodiode. (*b*) Back view of the Eiger X 9M, with all the signal and motor cables/air and water cooling lines carried by a cable drag chain as indicated.

The SAXS detector, Eiger X 9M, has a 500 Hz maximum frame rate and an active area of 233 × 245 mm. It sits on a detector stage with ten degrees of freedom, allowing vertical and horizontal translations of ±120 mm. In front of the detector is a 4 mm beamstop made of Ta (BS1), glued to the center of an 8 µm Kapton frame with ±20 mm translation capability in the vertical and horizontal directions [Fig. 4 ▸(*a*)]. Two additional beamstops, BS2 and BS3, closely spaced and each embedded with a PIN diode, are positioned near BS1. Both BS2 and BS3 are designed for grazing-incidence SAXS (GISAXS) to block the direct beam and possible strong strip scattering; they can be moved out of the detecting area during transmission-type SAXS measurements. BS1 and BS3 are moved horizontally out of and into the direct beam position for transmission measurements and incidence-angle determination in GISAXS, respectively. Position encoder systems comprising optical rulers are embedded in these two beamstops, ensuring no risk of exposing the detector to the strong direct beam as a result of possible missteps of the beamstop motors.

All cables of the Eiger X 9M detector, motor cables, power cords and other signal cables (15 m long) are arranged in a cable drag chain, allowing the detector to move freely on the rail system inside the detector vacuum vessel [Fig. 4 ▸(*b*)]. The detector usually travels at a cautious speed of 0.5 m min^−1^ for sample-to-detector distances (SDs) between 0.7 and 10.2 m. The encoder system constantly monitors the position of the detector, which is updated to the corresponding process variable (PV) in the integrated control system detailed below.

#### 3.3.3. WAXS detector and SDD

**Figure 5:**
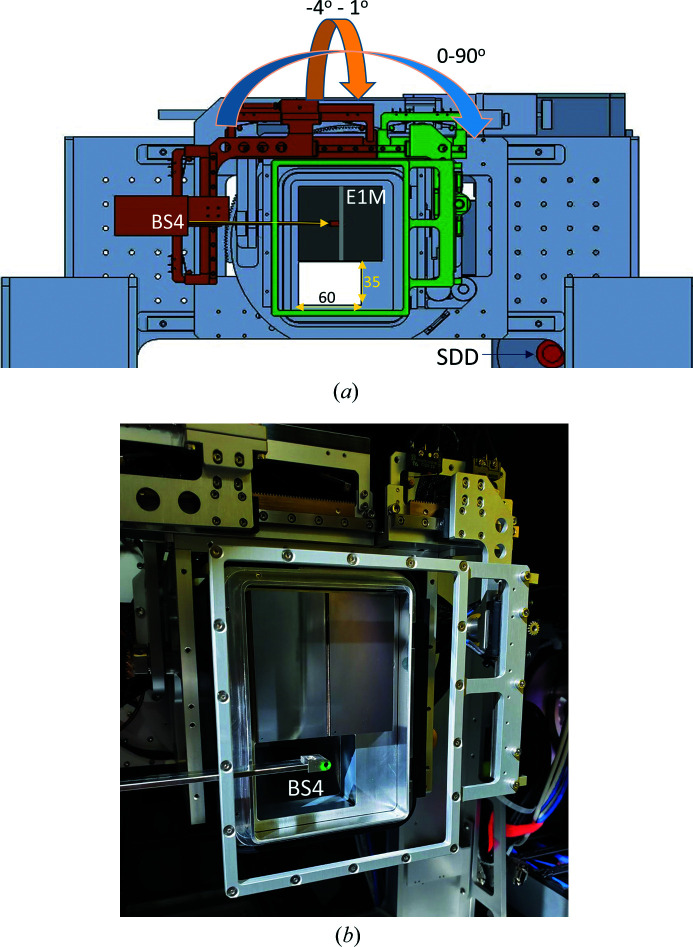
(*a*) Illustration of the Eiger X 1M (E1M) with an opening of 35 mm by 60 mm. The beamstop BS4 (orange) is also shown on the detector stage of multi-degrees of freedom (tilt, rotation, horizontal movement and vertical translation). Located at the bottom-right corner is a silicon drift detector (SDD, red pillar). (*b*) Photograph of the Eiger X 1M. The cable drag chain that carries all relevant wiring is on the right.

A custom-made Eiger X 1M detector (Dectris) with a maximum frame rate of 3 kHz is used as the WAXS detector at the TPS 13A endstation. The detector has a custom-designed opening of 35 × 60 mm only 1.2 mm from the edge of the active area (79.9 × 77.2 mm) for minimal blocking of the SAXS detector view angle in a simultaneous SWAXS measurement (Fig. 5 ▸). The detector stage offers translations of ±45 mm and −45 to 305 mm in the horizontal and vertical directions, respectively. Moreover, the detector can tilt −4–1° along the beam path and rotate by 0–90° perpendicular to the beam path [Fig. 5 ▸(*a*)]. The whole detector stage can translate on the same double-rail system that carries the SAXS detector stage. In SWAXS measurements, the direct beam bypasses the Eiger X 1M through a custom-made slot (Fig. 5 ▸) and falls on BS1. When Eiger X1 M is used alone to take a full-view (360° azimuthal angle) WAXS pattern, a motorized tungsten beamstop (BS4) equipped with a photodiode (Fig. S4) is installed to selectively move into the active area of Eiger X 1M to block the direct beam. The position alignment of BS4 to the direct beam is carried out by a diode intensity scan in the vertical and horizontal directions (via an auto-centering macro) with an attenuated direct beam.

All Eiger X 1M detection system cables are carried by a cable drag chain that can unfold or recoil with the translation of the Eiger X 1M along the X-ray path on the rail system [Fig. 5 ▸(*b*)]. With a regulated 0.5 m min^−1^ speed, the detector can move within an SD range of 0.18–9 m; the corresponding detector position is monitored by a position encoder and updated to the PV of the integrated control system. The shortest SD (0.18 m) is restricted by the space taken by the fast-closing shutter and its adaptor [Fig. 3 ▸(*d*)], which consumes nearly 100 mm of X-ray path length. We note that putting the WAXS detector in vacuum effectively minimizes air scattering for greatly enhanced WAXS data quality, although the detectable *q* range is inevitably restricted. Nevertheless, an often used WAXS configuration with a 15 keV beam and 0.3 m SD still can cover up to *q* = 2.5 Å^−1^ for solution samples.

An SDD (Amptek XR100-SDD) and two cameras are situated inside a box attached to the WAXS detector stage [Fig. 5 ▸(*a*)]. One camera monitors the signal lights on the back panel of the Eiger X 1M; the other camera checks the motion of the Eiger X 9M. The SDD is used to locate the sample position, monitor the beam energy and record scattering/fluorescence X-rays from the sample. The heat generated by the SDD is removed through a regulated airflow connecting to a branch of the endstation’s central dry air supply system, maintaining the box temperature below 313 K. The SDD has a 25 mm active area and can detect X-rays in a wide energy range covering 4–23 keV with an energy resolution of 110 eV. It is located just below the Eiger X 1M and 120 mm to the side of the center [Fig. 5 ▸(*a*)].

## 4. Endstation operation

### 4.1. Integrated control scheme

The TPS 13A endstation adopts the *Experimental Physics and Industrial Control System* (*EPICS*) for integrated control of the beamline hardware and software (Liu *et al.*, 2021 ▸), including all motors and sensors. *EPICS* can coordinate all the beamline and endstation components by saving and coding all the functional statuses of the beamline and endstation components as *EPCIS* PVs. Thereby, synchronized and coordinated operations of the beamline and endstation components can be performed through sequential or batched reading (output) or changing (input) of the *EPCIS* PVs, generated by input–output–controller server programs (Chiang *et al.*, 2019 ▸). There are two main clients of the *EPICS* PVs: (1) the graphical user interface named *Control System Studio* (*CSS*) and (2) the UNIX-based software package *SPEC* (SPEC Control Systems Ltd). Both can access and operate *EPICS* PVs to execute integrated operations of beamline components and data collection. Status displays and operations of the major endstation components are integrated into *CSS*, providing visual icons, status indicators and interactive dialog inputs for intuitive user operation of beamline/endstation components with successive inputs of PV values. On the other hand, *SPEC* macro scripts control all *EPICS* PVs to perform batched operations of the beamline/endstation components. The batched operation can combine different functions, including sequential movements of beamline components alternated with data collections and real-time data display with analysis. For example, the CEN-slit macro would open the JJ-SP2 slits, move in a photodiode, and scan the JJ-SP1 slits in the vertical and horizontal directions to allocate the beam position. The macro continues to move the JJ-SP1 slit opening to the beam position, center the JJ-SP2 slits and move the photodiode out of the X-ray beam path to complete the macro. The TM-macro for sample transmission measurement would read the beam-energy status (4–23 keV) and move in an auto-calculated combination of attenuation foils according to an assigned attenuation factor. The procedure is followed by moving out the beamstop from the direct beam, triggering the Eiger X 9M for exposure, moving back the beamstop and moving out all attenuation foils to return to the SAXS mode.

### 4.2. Synchronized sample controls and SWAXS data acquisition

**Figure 6:**
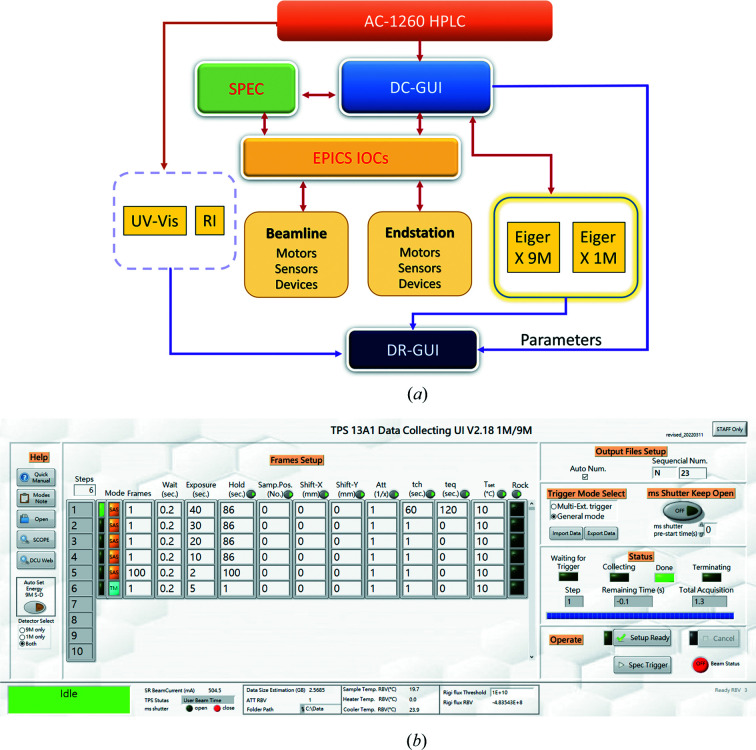
(*a*) SEC–SWAXS data collection scheme initiated by the SEC system. Red path arrows represent trigger directions, and blue path arrows show the data flow. The data collection GUI (DC-GUI) is initiated by the sample system (SEC system in SEC–SWAXS) and then executes a data collection plan through coordinating the X-ray detection system with the other endstation components (*e.g.* Rigi/Civi XBPMs and MS shutter). (*b*) The DC-GUI for programmable data collection strategies up to 999 steps. For each step (row), users can set either SAXS (SAS) or transmission mode (TM), the number of frames, the wait time (between two frames), the exposure time, the hold time (before the next step), the sample position, the shift of the predefined sample positions in the horizontal and vertical directions, the attenuation factor of the beam intensity (Att), the sample temperature, and the sample position rocking (to reduce radiation damage). Users can also choose which detector to use at the bottom-left corner. On the right-hand side, the millisecond shutter can be set to stay open during the whole data collection procedure or open only during the exposure time (to avoid unnecessary beam exposure on the sample). The table shows typical steps for SEC–SWAXS measurements.

Fig. 6 ▸(*a*) shows the designated trigger direction and data flow to conduct a SEC–SWAXS data collection at TPS 13A. The SEC–SWAXS data collection GUI (DC-GUI) can be triggered by the SEC system (HPLC/UV–Vis/RI) or *SPEC* to control the two Eiger X-ray detectors and the endstation components. Multiple data collection steps are developed on the DC-GUI to run batched SWAXS and transmission data collection [Fig. 6 ▸(*b*)]. For each step, the number of frames, exposure time, waiting times between exposures and between steps, sample temperature, and sample position (position on the capillary cell) can all be adjusted according to a specific data collection strategy. The MS shutter can be selectively closed during the waiting time between the data frames in each step and the holding time between steps to minimize unnecessary sample X-ray exposure. In a SEC–SWAXS experiment, the SEC system simultaneously triggers UV–Vis detection, RI measurements and the DC-GUI to run the programmed batched SEC–SWAXS data collection after initiating the auto-sampler in the SEC system. In principle, users would need to operate the two GUIs of X-ray data collection (DC-GUI) and the Agilent system (through the *VOYAGER* GUI under the *VISION* software hub of WYATT Technology Corporation, Santa Barbara, CA, USA). *VOYAGER* [detailed in Fig. S2(*a*)] defines all the Agilent 1260 instrument parameters and synchronizes the measurements of UV–Vis absorption, RI, MALS and DLS. With the DC-GUI, users can define all the data collection strategies with synchronized or decoupled Eiger X 9M and Eiger X 1M and the sample environment, including sample temperature and capillary exposure position, as illustrated in Fig. 6 ▸(*b*).

### 4.3. Data processing

**Figure 7:**
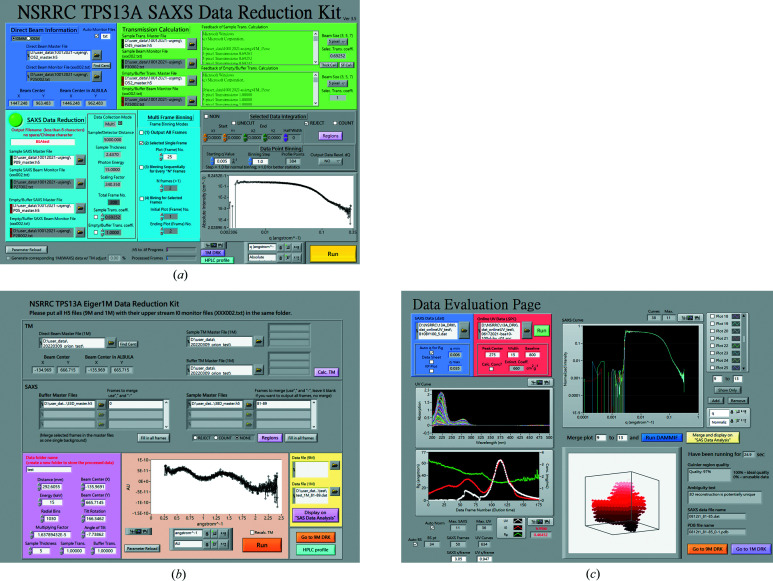
(*a*) The SAXS DR-GUI at TPS 13A, illustrated by a sample solution of 10 mg ml^−1^ BSA with 100 µl injection. The upper part is used for transmission calculation and the lower part for the Eiger X 9M SAXS data reduction. Users input the sample file name, select detector image files, choose one of four image binning modes and hit the ‘Run’ button to conduct the reduction of the 2D images to the circularly averaged 1D *I*(*q*) profiles displayed in the lower-right corner. Alternatively, line-cut profiles or ROI on a 2D pattern can be selected or rejected in the processing. (*b*) The parallel WAXS DR-GUI, operating independently of or collectively (sharing the same instrument parameters) with the SAXS DR-GUI on the simultaneously measured SWAXS data. (*c*) The data evaluation page (DEP) with the SEC–SAXS data and online UV absorption data measured at the same sample position over the sample elution. The DEP processes the data generated from the SAXS DR-GUI to calculate SAXS *I*(0) (bottom left, red curve) and *R*_g_ (green curve) and display them with the sample concentration deduced from the online UV–Vis absorption (at a selected wavelength) for chromatographic evaluation (white curve). The upper-right graph allows multi-profile comparison. The well overlapped data profiles with similar *R*_g_ can be merged and converted into a *p*(*r*) function by clicking the yellow button. A *DAMMIF* simulation can then be selectively executed, with results displayed in the bottom-right corner from an auto-preliminary data analysis.

We have developed a *LabVIEW*-based (National Instruments Corporation, Austin, TX, USA) data reduction graphical user interface (DR-GUI) [Fig. 7 ▸(*a*)] that allows integrated and high-throughput SWAXS data processing. The DR-GUI provides quick visual drag-and-drop of selected detector image files collected from each step in the DC-GUI. Once the input files are selected, the DR-GUI will automatically read the corresponding header file generated by the DC-GUI. The information file contains the beam intensity data measured by the Rigi and Civi XBPMs and other metadata, such as the direct beam coordinates, SD and beam energy. A default circular average is selected for isotropic scattering patterns to reduce the 2D patterns into 1D scattering intensity profiles *I*(*q*); region-of-interest (ROI) or line-cut profiles are also available. The corresponding WAXS data reduction can be processed independently or simultaneously with the SAXS data, sharing all the instrument parameters [Fig. 7 ▸(*b*)]. With a calibrated SWAXS alignment scaling factor (discussed below), the WAXS profiles can automatically align to the corresponding SAXS profiles. Other functions like data point binning, data resolution calculation, data image merging and ROI setting (*e.g.* rejection of the beamstop area or line/box integration along a specific direction) are also integrated into the DR-GUI.

Furthermore, the DR-GUI offers a data evaluation page [DEP, Fig. 7 ▸(*c*)], where all the SAXS *I*(*q*) profiles can be examined, compared and merged according to the chromatographic evaluation with the UV–Vis profiles, *I*(*0*) values and radius of gyration (*R*_g_) values. The DEP uses the *ATSAS* tool *autoRg* to determine the *q* ranges for *I*(0) and *R*_g_ calculations (Manalastas-Cantos *et al.*, 2021 ▸), including evaluating possible aggregate formation. An option for manually changing the *q* range for *R*_g_ determination is provided for weak scattering profiles. For SEC–SWAXS data, the DEP also converts the UV–Vis absorption data into a concentration evolution profile based on Beer’s Law (requiring inputs of a UV–Vis absorption coefficient). The concentration profile was plotted with *I*(0) and *R*_g_ along one HPLC sample elution, as shown in the lower-left corner of Fig. 7 ▸(*c*). All *I*(*q*) profiles are normalized to the profile of the frame with the highest *I*(0) and displayed in the DEP [Fig. 7 ▸(*c*), upper right] either in the log–log *I*(*q*) versus *q* format or in the Kratky–Porod (KP) representation. Well overlapped profiles can be identified and selected manually for further multi-frame binning to improve the data statistics. We have also integrated other data analysis functions in *ATSAS* (Manalastas-Cantos *et al.*, 2021 ▸) into the DEP, including (1) checking the Guinier region quality, (2) examining the KP plot for background subtraction quality or molten protein structures, (3) estimating data ambiguity, (4) generating distance distribution functions *p*(*r*), and (5) executing *DAMMIF* to obtain an *ab initio* model shape. All plots and results on the DEP shown in Fig. 7 ▸(*c*) can be exported to ASCII files. The 1D scattering intensity profiles *I*(*q*) with errors and *p*(*r*) can be submitted to other data analysis software.

**Figure 8:**
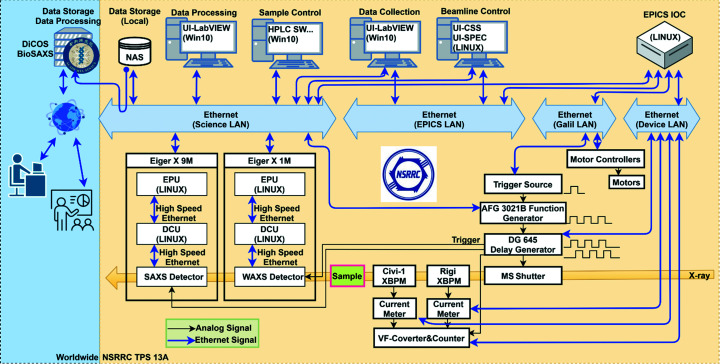
Ethernet network of the TPS 13A endstation. The digital signals are communicated via four Ethernet subnetworks: Science LAN (for data), EPCIS LAN (for beamline control), Galil LAN (for motors) and Device LAN (for others). All data are temporarily stored in a local NAS and updated to the DiCOS BioSAXS platform constructed by the Academia Sinica Grid Computing Centre (top-left corner) for storage, retrieval and processing.

All raw image files and processed data are temporarily saved to local network attached storage (NAS). The NAS is linked to the Distributed Cloud Operating System (DiCOS) BioSAXS platform developed by Academia Sinica Grid Computing Centre for further data processing and long-term data storage (Fig. 8 ▸). The platform syncs its file content with the endstation NAS every 15 min, with a DiCOS petabyte-scale data storage system and over 10 000 on-demand CPU cores. Through a web-based GUI, the DiCOS BioSAXS platform provides access to all registered users to retrieve data, run data reduction (or recalculate with modified parameters), and submit online jobs for running *DAMMIF*, *DAMMIN* and *GASBOR* using a cluster version *ATSAS* package (collaboration with the biological small-angle scattering group of the European Molecular Biology Laboratory). More software and the corresponding user interfaces for data analysis are currently under construction. The internal and external data communication network is based on four Ethernet clusters: Science LAN, EPCIS LAN, Galil LAN and Device LAN (Fig. 8 ▸).

## 5. Performance

### 5.1. Instrument calibrations

**Figure 9:**
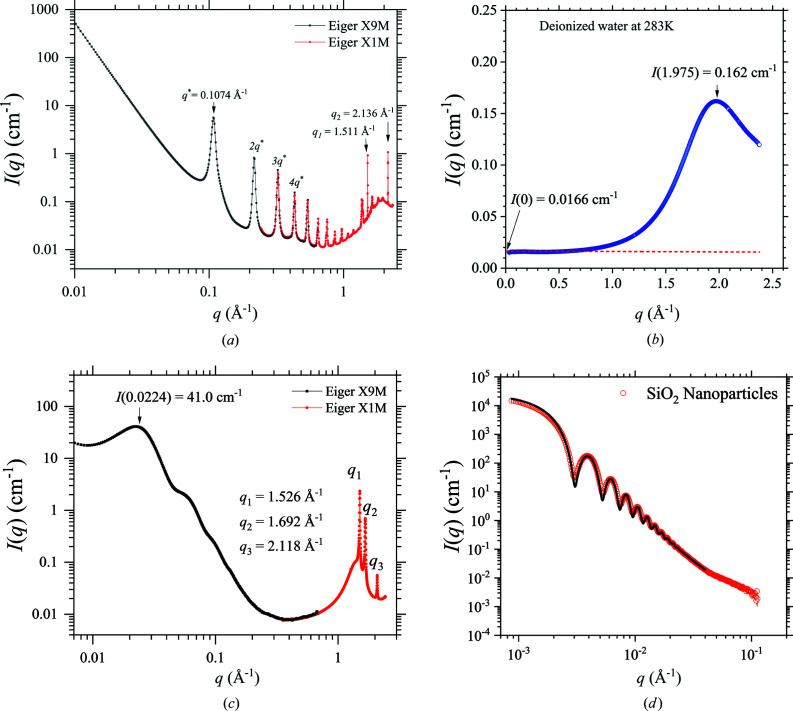
(*a*) Standard calibration of the SWAXS *q* value of the TPS 13A endstation using mixed powders of silver behenate (periodic peaks marked) and LaB_6_ standards (two characteristic peaks *q*_1_ and *q*_2_ indicated). An alignment factor is determined by scaling the SAXS data taken by Eiger X 9M and the WAXS data taken by Eiger X 1M. The factor is applied to all the subsequent SWAXS measurements. (*b*) SWAXS data of deionized water. The profile is scaled (with a scaling factor *f*) to reach an extrapolated (dotted line) *I*(0) value at 283 K, as indicated. The broad WAXS peak at *ca* 1.975 Å^−1^ corresponds to the water correlation length. (*c*) SWAXS data measured for high-density polyethyl­ene. The data are calibrated by the standard procedure of water scattering and can serve as a secondary standard for *q* position and absolute intensity, using the calibrated values indicated. (*d*) USAXS data of SiO_2_ particles in solution, using a 10 keV beam and SD = 10.0 m. The data are fitted (solid curve) with 5% polydisperse core–shell spheres with a mean core diameter of 284 ± 11 nm and a shell thickness of 3.6 nm (with a slightly lower scattering length density than the core).

The *q* value of the SWAXS data at the TPS 13A endstation is calibrated with the well defined SAXS and WAXS peaks simultaneously measured from mixed powders of the standards of silver behenate and lanthanum hexaboride (LaB_6_) [Fig. 9 ▸(*a*)]. From the peak positions of the standards, the SDs of the SAXS and WAXS detectors are determined. The image distortion correction in the WAXS region is carried out using *FIT2D* (Hammersley, 2016 ▸). We also measure water scattering to scale the SWAXS data [Fig. 9 ▸(*b*)] to the absolute intensity scale (cm^−1^) with a scaling factor *f*, following the procedure discussed previously (Jeng *et al.*, 2010 ▸). Moreover, the SWAXS data define an absolute intensity *I*(*q* = 1.975 Å^−1^) = 0.162 cm^−1^ for the temperature-dependent water correlation peak at 283 K, which might be useful in water density determination (Kim *et al.*, 2017 ▸). The calibrated SDs and the scaling factor are applied to a high-density polyethyl­ene with characteristic and stable small- and wide-angle peaks that can be used as a secondary standard (Jeng *et al.*, 2010 ▸) for convenient *q*-position and absolute intensity calibrations [Fig. 9 ▸(*c*)]. After the instrument calibration, further changes of SAXS and WAXS SDs due to detector translations inside the large vacuum vessel are automatically updated to the DR-GUI through the position encoder system and the PV names of the two Eiger detectors; this greatly reduces the effort on instrument *q* calibration due to changes in the detector positions. Moreover, large SiO_2_ nanoparticles in an aqueous solution were examined by our USAXS mode with *q* starting around 0.0008 Å^−1^; the fitted diameter of 284 nm [Fig. 9 ▸(*d*)] demonstrates the USAXS capability of the endstation in solution scattering (Narayanan *et al.*, 2018 ▸). With a 6 keV beam and a sample-to-detector distance of 10.0 m, the lowest achievable *q* reduces to 0.0004 Å^−1^; the first peak at *q* = 0.000628 Å^−1^ of a gold-array standard of 1 µm *d* spacing could be observed with a peak width of *ca* 0.00004 Å^−1^ (Fig. S5), demonstrating the USAXS resolution at the peak position.

### 5.2. Model proteins

**Figure 10:**
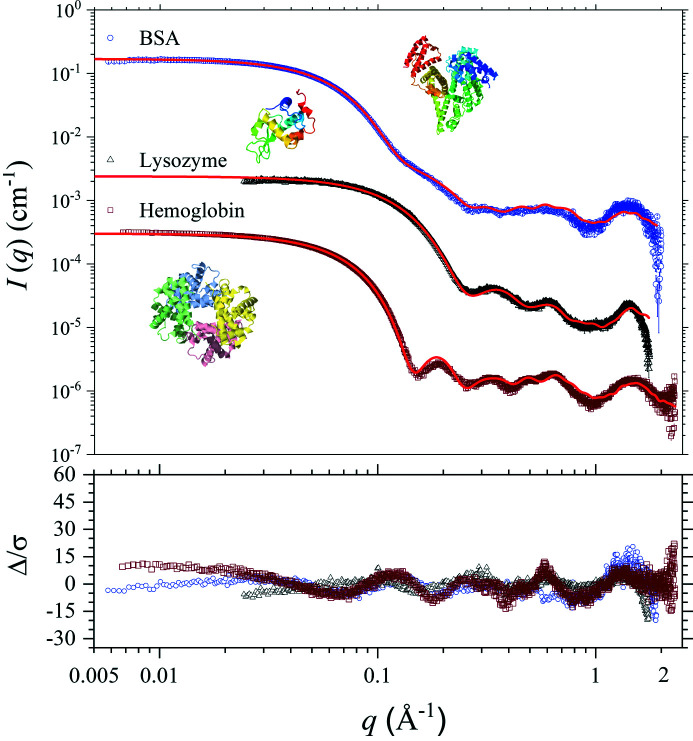
SWAXS data measured at TPS 13A for BSA (top), lysozyme (middle) and hemoglobin (bottom); also shown are the corresponding crystal structures. The sample concentrations are all 10 mg ml^−1^. With 100 µl injection volume, the peak sample concentration during the HPLC elution reduces to *ca* 4 mg ml^−1^ (*cf*. the dilution factors shown in Fig. S6). The WAXS data of BSA (Fig. S7) are replaced by those measured with the bypass mode (of little sample elution dilution effect) for better statistics. The intensity profiles of lysozyme and hemoglobin are, respectively, scaled down from the absolute intensity scales by factors of 50 and 500. Also shown are the fitting curves calculated using *CRYSOL* (dotted red curves) based on the crystal structures with PDB ID 3v03 for BSA, 1lyz for lysozyme and 1a3n for hemoglobin. The lower inset shows the fitting residual Δ/σ defined by the differences (Δ) of the model and experimental results normalized by the corresponding data uncertainties or errors σ (Trewhella *et al.*, 2017 ▸).

SEC–SWAXS was performed with the sample solutions of a few model proteins, including lysozyme from chicken egg white (14.3 kDa), human hemoglobin (64.5 kDa) and bovine serum albumin (BSA, 66.5 kDa), to illustrate the capability of the TPS 13A endstation. As shown in Fig. 10 ▸, the SWAXS data are merged from the data simultaneously collected with the SAXS and WAXS detectors during the same HPLC sample elution, covering a dynamic *q* range (*q*_max_/*q*_min_) larger than 100. An additional data collection with the bypass mode (avoiding the sample dilution effect with SEC–SAXS, as shown in Fig. S6) was performed for the same BSA sample solution to improve the data statistics in the intermediate *q* region (0.2–0.6 Å^−1^) which exhibits fine features. For the smaller protein, lysozyme, *R*_g_ can be extracted with the Guinier approximation in a relatively higher *q* range that still satisfies the criterion *q* < 1.3/*R*_g_ (Guinier & Fournet, 1955 ▸). Therefore, the SD was shortened to 2.0 m for SAXS from 3.5 m used for the other two larger proteins to enhance the middle *q* region (0.2–0.6 Å^−1^). Correspondingly, the WAXS detector was tuned to 0.6 m from 0.3 m. The three sets of data could cover the respective low-*q* regions needed to extract the *R*_g_ values of 15.1 ± 0.1 Å for lysozyme, 25.07 ± 0.02 Å for hemoglobin and 28.2 ± 0.2 Å for BSA, using the Guinier approximation. Moreover, pronounced humps measured in the WAXS region (0.3–2.0 Å^−1^) reveal prominent local structural features of the proteins. These SWAXS data were measured using a 15 keV beam (with DCM) and flux of ∼1 × 10^12^ photons s^−1^ at the sample area. For all three proteins, 100 µl of sample (10 mg ml^−1^) were injected into the SEC system thermostated at 283 K with a flow rate of 0.35 ml min^−1^. The data collection frame rate was 2 s per frame over the sample elution peak; buffer scattering was collected before and after the elution peak for background subtraction (as detailed in Section S8 of the supporting information). Sample scattering data were merged from the ten best-overlapped frames for a circularly averaged profile. After each sample run, a routine washing sequence comprising 2 min of deionized water (DIW) flow, followed by 3 min of Hellmanex III flow, then 3 min of DIW flow, could efficiently remove damaged protein deposited on the X-ray irradiation spot of the sample capillary (Fig. S3).

Fig. 10 ▸ compares the data with the scattering profiles calculated from the corresponding crystal structures [BSA PDB ID 3v03 (Majorek *et al.*, 2012 ▸), lysozyme PDB ID 1lyz (Diamond, 1974 ▸) and hemoglobin PDB ID 1a3n (Tame & Vallone, 2000 ▸)] using *CRYSOL* (Franke *et al.*, 2017 ▸). For lysozyme, the calculated profile matches very well with the SWAXS data, including the two prominent peaks around 0.35 Å^−1^ (a form factor feature) and 0.6 Å^−1^ (*ca* 10 Å characteristic distance dominated by interactions between α helices) (Phan-Xuan *et al.*, 2020 ▸). The seamless fit up to *q* ≃ 2.0 Å^−1^ for a spatial resolution of a few ånsgtröms opens new horizons of using SWAXS in validating atomistic models of biomolecules proposed with different methodologies. The scattering profile calculated on the basis of the BSA crystal structure can describe our SWAXS data well throughout the wide *q* range (*cf*. SASDA32 data in the Small-Angle Scattering Biological Data Bank at https://www.sasbdb.org/). The result suggests that the relatively large multi-domain protein could still hold a steady conformation against thermal fluctuations in solution for nearly the same structural features as predicted from the crystalline form. Even the tiny multi-peaks in the middle *q* region (0.4–0.8 Å^−1^) in the calculated profile match well to the measured humps (Fig. 10 ▸). The intramolecular correlations are much more complicated for the hemoglobin case due to a quaternary configuration formed by four symmetric units (Cammarata *et al.*, 2008 ▸). Correspondingly, richer scattering features are observed in the middle to high *q* region. The calculated profile follows the several feature humps closely, with the fitting residual shown in Fig. 10 ▸. There are some intensity deviations in the middle *q* region, which might be associated with the local structures of the quaternary configuration of the protein in solution being more mobile than in the crystalline form.

With general data statistics, less radiation damage and more tolerance in timing the elution peak for X-ray exposure, the protein SEC–SWAXS measurement configuration at TPS 13A (for thermal equilibrium conformations) is optimized to a 15 keV beam of a flux of *ca* 1–2 × 10^12^ photons s^−1^ (DCM mode), a sample elution flow rate of 0.2–0.4 ml min^−1^, a sample temperature of 283–288 K, and a data collection frame time of 1–2 s for general protein solutions of a few mg ml^−1^ and an injection volume of 20–50 µl. The data collection *q* range is changed mostly by translating the highly mobile SAXS detector in vacuum for the wide SD range of 0.7–10 m (*q*-range scaling factor of 70); a further change in X-ray energy (4−23 keV) allows another *q*-range scaling factor of 6. Occasionally, we observed protein aggregation due to radiation damage, especially when testing with intrinsically disordered proteins. Proteins with improper buffers tend to form aggregates after the first few frames of data collection, for example, BSA in H_2_PO_4_ or KCl buffers. In contrast, BSA in a buffer solution of 50 m*M* HEPES (Gupta *et al.*, 2015 ▸) can withstand the typical TPS 13A SEC–SWAXS measurements and is routinely used in the instrument calibration. At a higher flux of *ca* 6 × 10^13^ photons s^−1^ (DMM mode) with a 13 keV beam, we found that even relatively stable cytochrome c (10 mg ml^−1^) showed aggregation in the first second of exposure with a 0.35 ml min^−1^ flow rate, followed by successively increased *R*_g_ values over a multi-frame data collection.

## 6. Conclusion

With the highly mobile and synchronized two-detector system in vacuum, the TPS 13A endstation has demonstrated a new trend of biomolecular solution scattering with a wide *q* range. The endstation provides a versatile environment that can be used to conduct measurements of biomolecular solutions for structure and composition determinations in one sample elution. The SWAXS data collection is standardized through a user-friendly data collection GUI. Complementarily, the developed data processing kit with its data reduction GUI allows batched, combined SWAXS data reductions and preliminary evaluation, as well as high-throughput data processing. The SWAXS measured for several model proteins in a wide *q* range illustrates fine structural features that were hard to observe before without a WAXS detector in vacuum. With all the features presented, the TPS 13A BioSAXS endstation provides new opportunities for the structural research of biomolecules, particularly in correlating the local and global structural changes.

## 7. Related literature

The following additional reference is cited in the supporting information: Hura *et al.* (1999 ▸).

## Supplementary Material

Supporting information file. DOI: 10.1107/S1600576722001923/ge5116sup1.pdf
