# Supplementary material for: Performance of the new biological small- and wide-angle X-ray scattering beamline 13A at the Taiwan Photon Source
Source: J Appl Crystallogr. 2022 Mar 18;55(Pt 2):340–52. doi: 10.1107/S1600576722001923 (PMC8985603; doi:10.1107/S1600576722001923)
Supplement: Supplementary file 1 [file j-55-00340-sup1.pdf]

JOURNAL OF
APPLIED
CRYSTALLOGRAPHY

Volume 55 (2022)

Supporting information for article:

Performance of the new biological small- and wide-angle X-ray scattering beamline 13A at the Taiwan Photon Source

O. Shih, K.-F. Liao, Y.-Q. Yeh, C.-J. Su, C.-A. Wang, J.-W. Chang, W.-R. Wu, C.-C. Liang, Y. Lin, T.-H. Lee, C.-H. Chang, L.-C. Chiang, C.-F. Chang, D.-G. Liu, M.-H. Lee, C.-Y. Liu, T.-W. Hsu, B. Mansel, M.-C. Ho, C.-Y. Shu, F. Lee, E. Yen, T.-C. Lin and U. Jeng

Supporting information

Performance of the new biological small- and wide-angle X-ray scattering beamline 13A at the Taiwan Photon Source

Authors

O. Shih,[a] K.-F. Liao,[a] Y.-Q. Yeh,[a] C.-J. Su,[a] C.-A. Wang,[a] J.-W. Chang,[a] W.-R. Wu,[a] C.-C. Liang,[a] C.-Y. Lin,[a] T.-H. Lee,[a] C.-H. Chang,[a] L.-C. Chiang,[a] C.-F. Chang,[a] D.-G. Liu,[a] M.-H. Lee,[a] C.-Y. Liu,[a] T. W. Hsu,[a] B. Mansel,[a] M.-C. Ho,[b] C.-Y. Shu,[c] F. Lee,[c] E. Yen,[c] T.-C. Lin,[d] and U. Jeng[a,d,*]

[a]National Synchrotron Radiation Research Center, Hsinchu Science Park, Hsinchu 30076, Taiwan; [b]Institute of biochemical sciences & Institute of biological chemistry, Academia Sinica, Nankang, Taipei 11529, Taiwan; [c]Academia Sinica Grid Computing Centre, Academia Sinica, Nankang, Taipei 11529, Taiwan; [d]Department of Chemical Engineering, National Tsing Hua University, Hsinchu 30013, Taiwan
[*]Corresponding author: usjeng@nsrrc.org.tw

IMPORTANT: this document contains embedded data - to preserve data integrity, please ensure where possible that the IUCr Word tools (available from http://journals.iucr.org/services/docxtemplate/) are installed when editing this document.

S1. Beamline layout

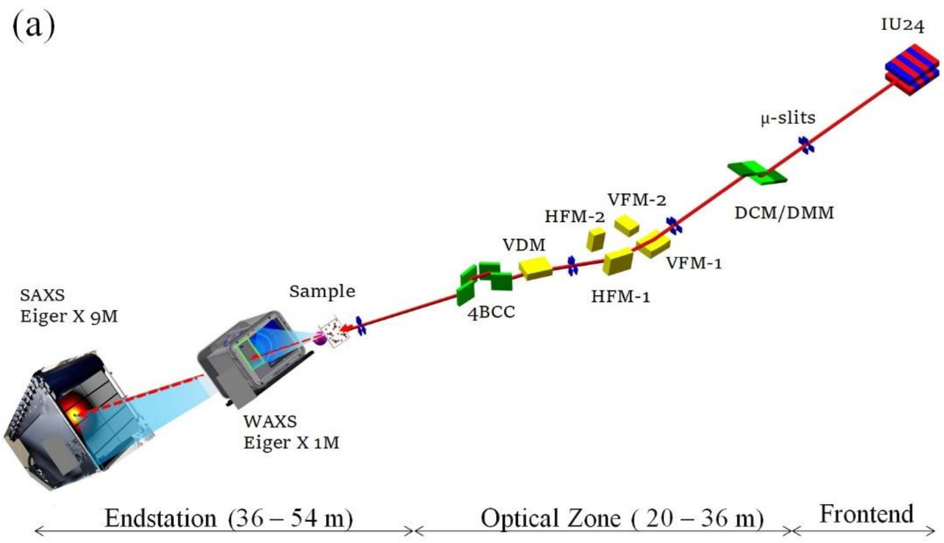

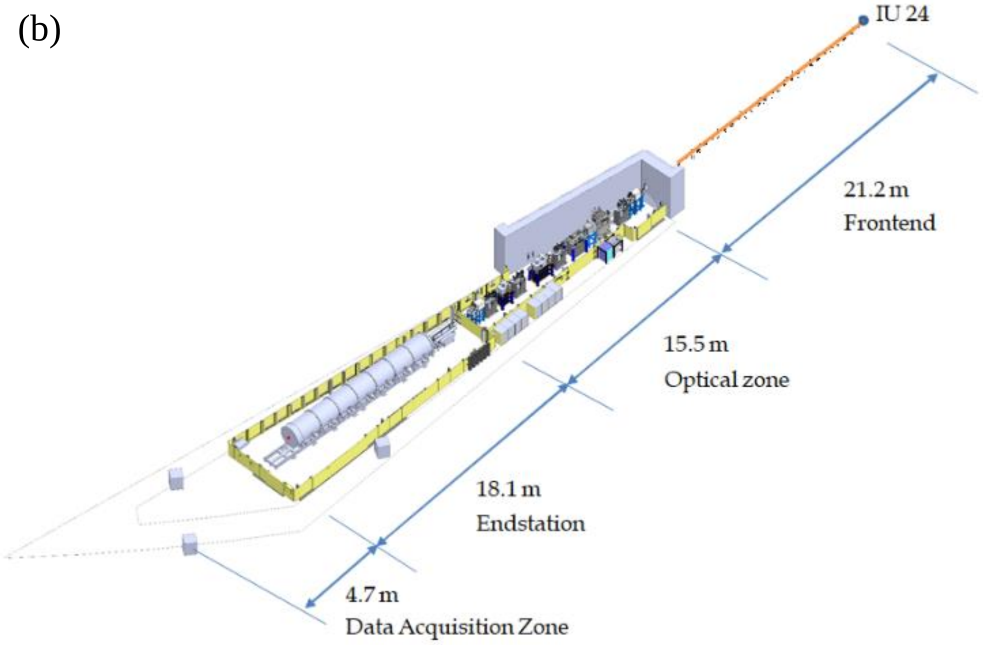

Figure S1 (a) 3D drawing of the primary optical components and beam path of the TPS 13A BioSAXS beamline. Starting from the right are the undulator IU24, microslits, DMM/DCM, two parallel sets of vertical/horizontal K-B focusing mirrors (VFM 1-2 and HFM 1-2), vertical deflecting mirror (VDM), and four-bounce double-crystal collimator (4BCC). Located in the endstation zone are the sample stage and the detecting system comprising Eiger X 1M for WAXS and Eiger X 9M for SAXS data collections. (b)The layout of the TPS 13 beamline, starting from the in-vacuum undulator (IU24) in the front-end, followed by the optical zone, endstation hutch, and the data acquisition zone.

S2. VOYAGER™ GUI for Integrated operations of Agilent 1260, UV-Vis, RI, MALS, and DLS

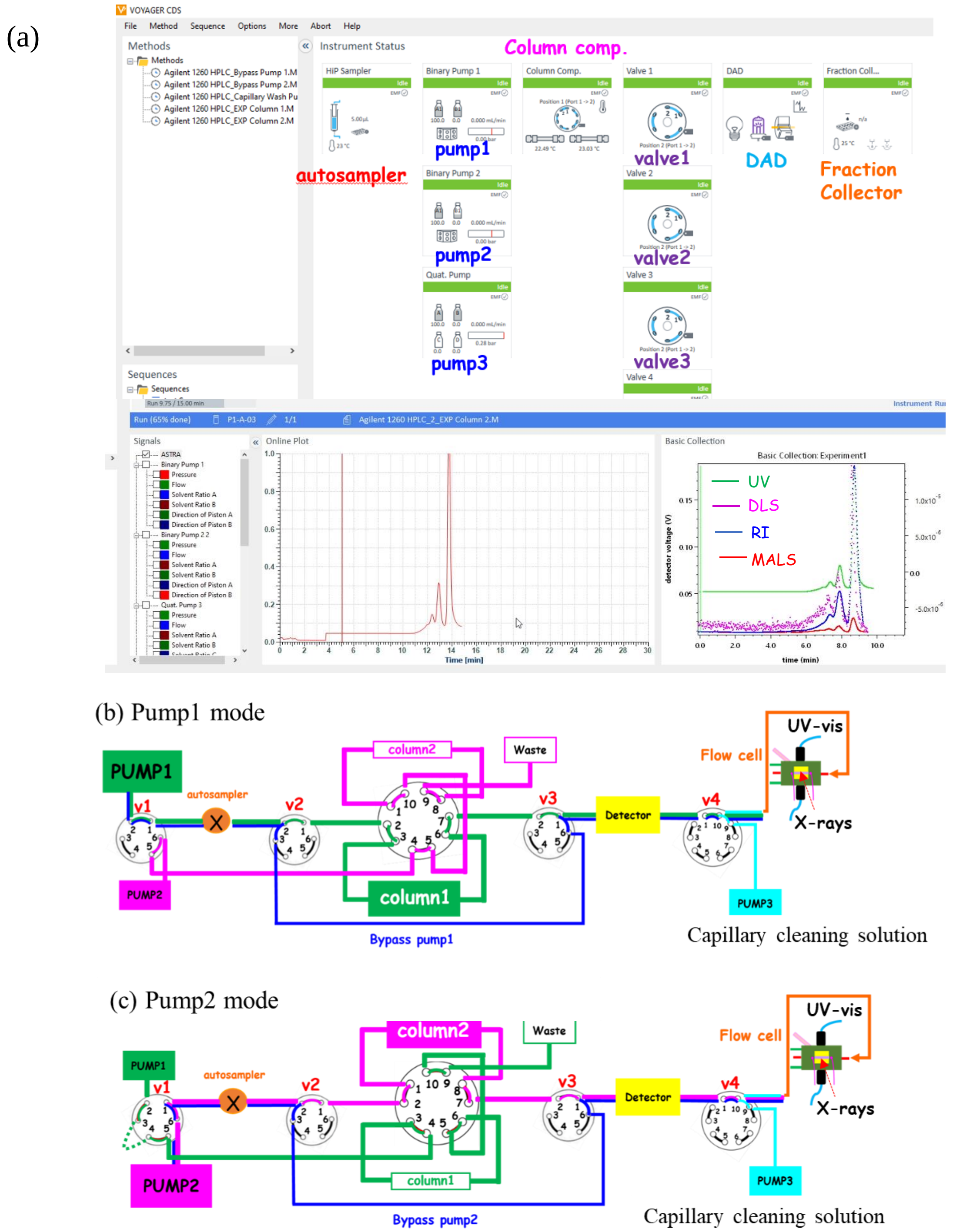

Figure S2 (a) Upper-half shows the VOYAGER™ GUI (under the VISION software hub of WYATT Technology Co.) used at the TPS 13A BioSWAXS beamline that integrates the Agilent 1260 HPLC operation and data collections of the UV-vis absorption, refractive index, multi-angle laser light scattering (MALLS), and dynamic light scattering (DLS); lower-half shows the data analyzed by the ASTRA GUI of VISION. (b) HPLC pump1 mode with column1 in duty while column2 in cleaning, and (c) Pump2 mode with column2 in duty, while column1 in cleaning. Either pump1 or pump2 can drive the bypass path (blue lines). The three pumps (pump1-3), four 6-way valves (v1-v4), and one 10-way valve (at the center) are marked. The capillary cell cleaning path is shown to the right-hand side with pump3. An online sample-mixing mode is also available.

S3. Sample capillary washing sequence with the SEC/UV-Vis/RI system

For a typical SEC-SWAXS experiment at the TPS 13A endstation, a preliminary run using 3 µL sample solution and a 0.35 mL/min flow rate is often conducted to determine the sample elution peak position before a production run. In a production run, the sample injection volume (20-100 µL) and flow rate (0.1 – 0.4 mL/min) depends on the sample conditions (centration, sample volume available, and the sample stability to X-ray irradiation). In general, the first production run is conducted with 100 µL and 0.35 mL/min; for improving data statistics, this could be followed by another production run with a slower flow rate around the elution peak for increasing data collection time/frames (typically 2 s/frame). A washing sequence for sample capillary using DIW and Hellmanex™ III (Hellma) is applied after each production run. Specifically, the washing sequence starts with 2 min of DIW flow, followed by 3 min of Hellmanex™ III flow, then 3 min of DIW flow in the end. Such washing procedure proves to be very efficient in removing sample deposition on the capillary spot where the X-ray beam irradiates (Fig. S3).

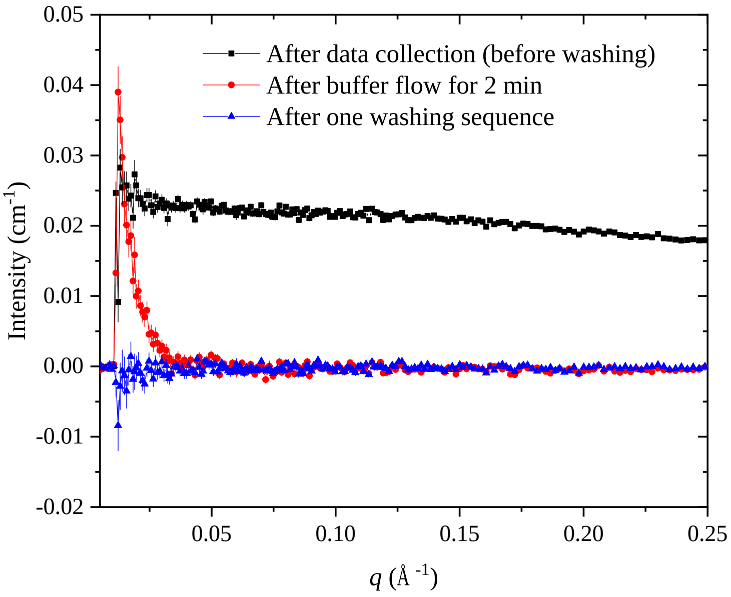

Figure S3 The SAXS profiles of the sample capillary at the TPS 13A endstation collected before, during, and after a standard washing sequence. The results illustrate different stages and degrees of residual/damaged protein deposition on the sample capillary, including (1) right after sample data collection (black), (2) after 2 min of buffer flowing (red), and (3) add 3 min of Hellmanex™ III flow followed by 3 min of DIW flow. After one washing sequence, the capillary shows no trace of protein deposition.

S4. Eiger X1M and X 9M with beamstops

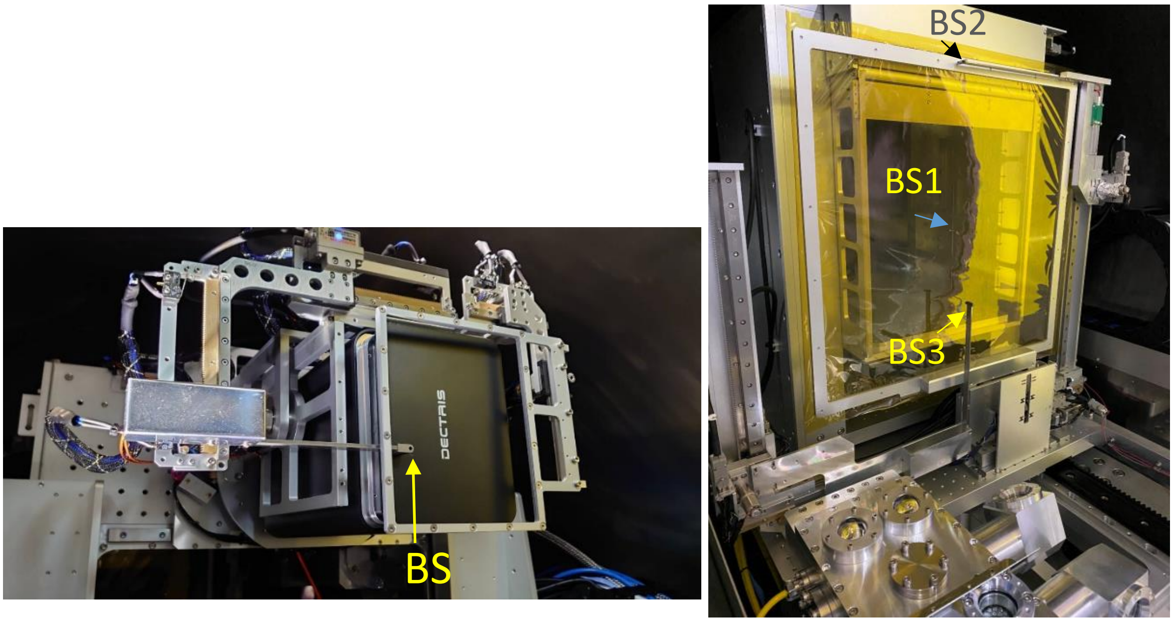

Figure S4 Left) Motorized BS4 of Eiger X 1M detector on the detector stage. BS4 is equipped with a photodiode, and can translate for ±50 mm in vertical and horizontal directions. Right) Front view of the Eiger X 9M and the three beamstops BS1, BS2, and BS3. Note that either BS2 or BS3 is with a photodiode.

S5. USAXS for gold arrays

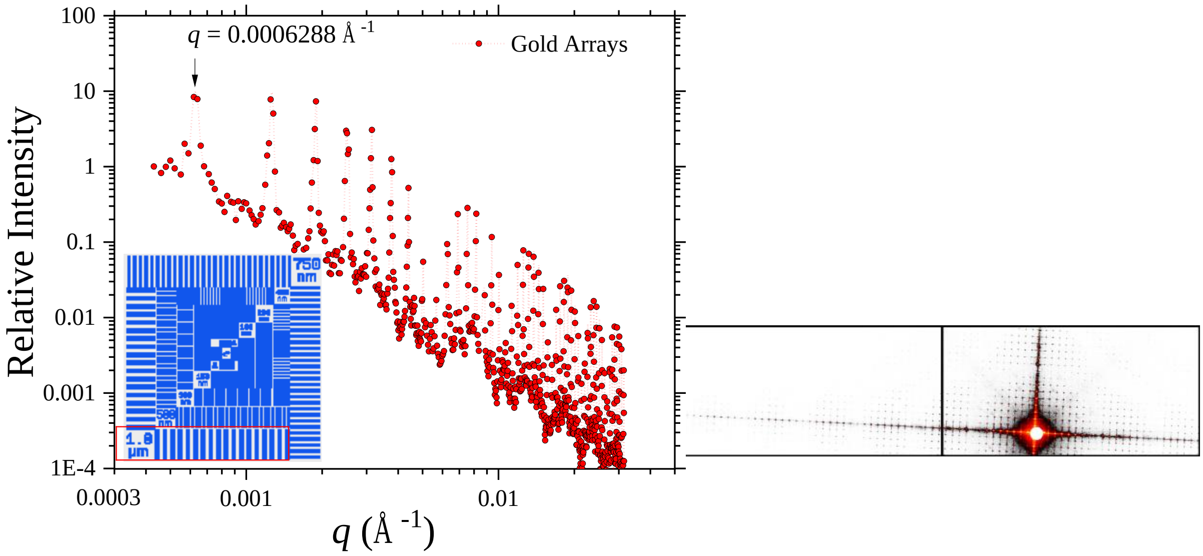

Figure S5 USAXS profile of a standard pattern of gold arrays (inset) extracted from the 2D pattern (on the right-hand-side) measured using a 6 keV beam and a sample-to-detector distance of 10.0 m. The arrow marks the first SAXS peak at q = 0.0006288 Å $^{-1}$, corresponding to the 1.0 µm pattern (red square in the inset). The peak width (~0.00004 Å $^{-1}$) reveals the q-resolution of the USAXS mode at the peak position.

S6. Sample dilution factor with the SEC-SAXS experiments

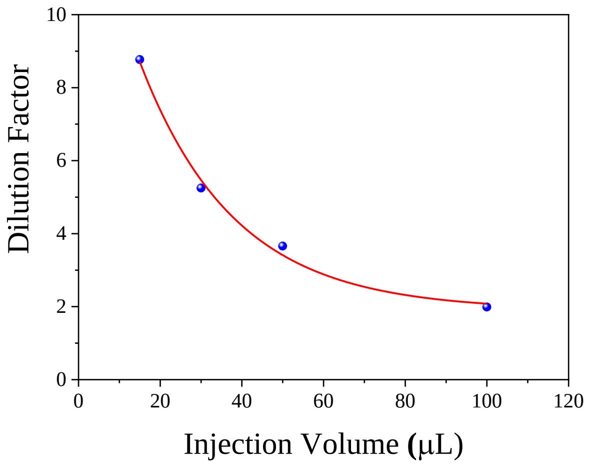

Figure S6 Dilution factor of sample concentration as a function of sample injection volume in the SEC-SWAXS measurements of the TPS 13 BioSWAXS endstation. The data were measured with an Agilent Bio SEC-3 silica-based column (pore size:300 Å).

S7. BSA SWAXS data

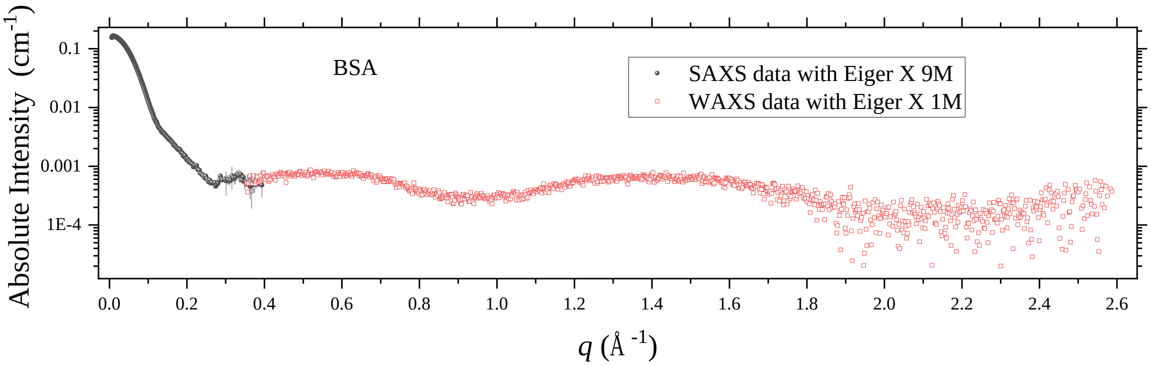

Figure S7 SWAXS data merged from the simultaneously measured SAXS data and WAXS data for BSA (10 mg/mL), with 100 μL injection volume and 0.35 mL/min flow rate.

S8. Background subtraction for SEC-SWAXS measurements

For protein solutions, SWAXS intensity in the middle to high-*q* region is generally weak, leading to critical importance in subtracting buffer scattering signal. With the SEC-SWAXS established at the TPS 13A endstation, the same capillary spot is used for the sample and buffer solution SWAXS data collections. This setup reduces the uncertainties in buffer intensity subtraction, due to inhomogeneity in capillary thickness. Moreover, we measured the two transmission values of a BSA sample solution (10 mg/mL) and its buffer, T_{sam} and T_{buff}, in the bypass mode, which were found to be undistinguishable.

Both values, $T_{sam} = T_{buff} = 0.700 \pm 0.003$, were measured with a 15 keV beam and 2.0 mm sample path length, using Eiger X 9M (to adequately select the direct beam zone on the area detector for an integrated direct beam intensity). For most protein solutions of concentrations up to a few tens mg/mL, the transmissions calculated using the X-ray linear absorption coefficient of BSA also indicate that the sample with the concentration contributes less than 0.1% difference in transmission under the same experimental condition. Therefore, the packaged SEC-SWAXS measurement procedure at the TPS 13A endstation includes only the transmission measurement of the buffer, but not the sample solution, which is designated to be conducted in the early stage of the HPLC elution before the sample elution peak. In the subsequent data reduction, the sample transmission is assumed to be the same as the buffer in the buffer scattering subtraction procedure; namely, the net intensity

$$I(q) = I_{sam}(q) - (T_{sam.}/T_{buff.})I_{buff}(q) \qquad (1)$$

with the T_{sam} in (1) approximated by the T_{buff} value measured. Here, $I_{sam}(q)$ and $I_{buff}(q)$ are the pixel intensities at the q value measured with the sample and buffer solutions, respectively. We found that this approximation works well in general, and there is no observable over-subtraction issue up to $q \sim 2.0$ Å^{-1} (main text, Fig. 10). Consistently, the measured buffer transmission is used as the sample transmission in the absolute intensity scaling procedure of the SEC-SWAXS.

For certain cases with strong hydration interactions, the $T_{sam} = T_{buff}$ subtraction approximation would yield minor over-subtraction in the high-q region near the water peak centered ca. 2.0 Å^{-1}, possibly owing to a shifting of the water scattering peak (cf. Fig. 9b); the water peak shifting might associate with changing of protein bound-water structures (Hura *et al.*, 1999). In such cases, an empirical buffer transmission adjustment within 0.25% (which is about the transmission resolution of the TPS 13A BioSWAXS instrument) in (1) is often enough to counteract the over-subtraction. Certain beamlines carry out buffer subtraction by matching the heights of the water peak of the buffer and the protein solutions; a similar adjustment in the scaling ratio is adopted to account for the over-subtraction issue in WAXS region (Yang *et al.*, 2020).

References.

Hura, G., Sorenson, J. M., Glaeser, R. M. & Head-Gordon, (1999) T. *Perspect. Drug Discov. Des.* **17**, 97-118.

Liu, D.-G., Chang, C.-H., Chiang, L.-C., Lee, M.-H., Chang, C.-F., Lin, C.-Y., Liang, C.-C., Lee, T.-H., Lin, S.-W., Liu, C.-Y., Hwang, C.-S., Huang, J.-C., Kuan, C.-K., Wang, H.-S., Liu, Y.-C., Tseng, F.-H., Chuang, J.-Y., Liao, W.-R., Li, H.-C., Su, C.-J., Liao, K.-F., Yeh, Y.-Q., Shih, O., Wu, W.-R., Wang, C.-A. & Jeng, U. (2021) *J. Synchrotron Rad.* **28**, 1954-1965

Yang, L., Antonelli, S., Chodankar, S., Byrnes, J., Lazo, E., Qian, K. (2020) *J. Synchrotron Rad.* **27**, 804–812.
